# Review of Sanitary Measures

**Published:** 1895-01

**Authors:** 


### REVIEW OF SANITARY MEASURES.

THE State Cattle Commissioners of Massachusetts have just completed their annual report, and it contains much rich food for thought and consideration. Under the present law some 394 inspectors have been employed, 136 of whom are farmers, 56 veterinarians, 10 cattle dealers, 8 butchers, 20 doctors of medicine, 14 health officers; while the remainder, 132 in number, are not connected directly with either cattle or the public health. These various members are assigned districts for inspection of all contagious diseases and all carcasses of animals slaughtered under this act. Neat cattle are inspected semi-annually, March and October. The total number of cattle in the State reaches 233,536.

Previous to October 4, 1894, a physical inspection only was made. Since that time tuberculin has been used for the better detection of tuberculosis. Up to December 15, 1894, 164 town inspectors had reported 39,072 examinations, with 236 cases of tuberculosis. In using tuberculin skilled men only were employed, numbering 33.

In Nantucket out of 665 animals six were found diseased, and all of these were of animals imported from the main land or had been in cohabitation with such animals.

The report recites in detail the work at Brighton and Watertown markets, and says that, whether or not, in view of the circumstances under which the animals have to be examined, the tuberculin test can accomplish its best results. Conditions of excitement incident to recent travel, novel surroundings, etc., complicate the work. When all conditions are under control of the examiner, the temperature chart shows regular and consecutive results which can be relied on, but, with the manifold number of unseen conditions which may come in under the necessary conditions at these markets, complete success can hardly be expected. Such errors as have occurred have resulted from these unappreciable conditions. No other board has had experience from week to week as a guide. The result of the work for the first six weeks is as follows:

| | Nov. 21. | Nov. 28. | Dec. 5. | Dec. 12. | Dec. 19. | Dec. 26. |
|---|---|---|---|---|---|---|
| Examined | 270 | 187 | 154 | 308 | 303 | 170 |
| Condemned | 26 | 25 | 16 | 18 | 14 | 11 |
| Tuberculous | 23 | 18 | 8 | 17 | 13 | 10 |
| Not diseased | 3 | 7 | 8 | 11 | 1 | 1 |
| Held for retesting | 1 | 1 | 3 | 5 | 19 | 10 |

This table shows 6.21 per cent. of diseased animals, and that in judging 1432 animals twenty-one mistakes have been made; that fewer errors occurred during the last three weeks than in the earlier experiences. Under the conditions controlling the board does not consider this result discouraging. The general summary of work is as follows: Whole number of cattle reported by inspectors and others, 3295; whole number examined at markets, 1432; whole number examined at Nantucket, 665; total, 5392.

All these were tested with tuberculin, and there were found to be tuberculous: From inspectors and others, 810 or 24.58 per cent.; from markets, 89 or 6.21 per cent.; from Nantucket, 6 or 0.9 per cent.

It should be remembered that the inspectors' cattle were all suspects on physical examination. The market cattle probably furnish a fair representation of the condition of cattle in the State.

After discussing the tuberculin test the report concludes that the test may be relied upon, provided the necessary care has been observed. Criticisms as to its unreliability are discussed at some length, and associated with this is a widely distributed collection of indorsements of its use by scientists; and they further say that, after noting so many errors from only a physical examination, it is not apparent why tuberculin should not be used because it occasionally results in an error in diagnosis. They comment on the absence of reliable statistics to show the prevalence of the disease, and refer to the impossibility of accomplishing this before the use of tuberculin was adopted.

The use of milk from tuberculous cows is strongly disapproved, even though the disease may be in its primary stages. They state that no practicable test can be applied to milk, as it can only be purified by purifying the herd. The question of milk being sent in from herds outside of the State is referred to and commended to the attention of the Legislature.

One hundred and sixty cases of glanders have been examined and condemned.

A small outbreak of hog cholera, involving twenty-five herds, is also reported.

In connection with the commission they have established a laboratory for microscopical assistance in their work and the production of tuberculin.

The report discusses the question of the compensation by the State to owners of animals killed for infectious diseases, presenting the arguments for full compensation and the considerations that may be urged against it. It recommends full compensation to cattle owners in cases of condemnation for tuberculosis during the present campaign of extermination.

